# The Being Taken Seriously Questionnaire—Development and Psychometric Evaluation of a PREM Measure for Person-Centeredness in a High-Tech Hospital Environment

**DOI:** 10.3390/ijerph17082660

**Published:** 2020-04-13

**Authors:** Anna Forsberg, Andreas Rantala

**Affiliations:** 1 Department of Health Sciences, Lund University, SE-221 00 Lund, Sweden; anna.forsberg@med.lu.se; 2 Department of Cardiothoracic Surgery, Lund University, Skåne University Hospital, SE-222 45 Lund, Sweden; 3 Emergency Department, Helsingborg General Hospital, SE-205 01 Helsingborg, Sweden

**Keywords:** PREM, person-centered care, person-centeredness and high-tech environments

## Abstract

There is a need for patient-reported experience measures (PREMs) in high-tech hospital environments based on the philosophy of person-centeredness. The aim of this study was to describe the development and initial psychometric evaluation of a measure of person-centeredness by means of being taking seriously. In this cross-sectional survey, the study sample (n = 79) completed two measures, the Being Taken Seriously Questionnaire—Patient version (BTSQ-P) which was the measure undergoing psychometric evaluation, and the Person-Centered Climate Questionnaire—Patient version (PCQ-P) that was used to evaluate the climate in which the intervention was occurring. The expected scale dimensionality was examined both by the confirmatory multi-trait analysis program and by explorative principal component analysis (with oblique, varimax rotation). Scale reliability was estimated using ordinal alpha and Cronbach’s alpha. One solid factor was generated. This factor had good internal convergent validity and good item–scale reliability, covering 80.41% of the variance. The interitem correlation ranged from 0.759 to 0.908 and the alpha was 0.93 (ordinal alpha) and 0.87 (Cronbach’s alpha) respectively. There was a strong relationship between BTSQ-P and the PCQ-P dimension safety climate. In conclusion, the internal consistency, reliability and concurrent validity of the BTSQ-P was satisfactory for use in a high-tech hospital environment.

## 1. Introduction

Person-centered care (PCC) is the preferred approach to all patients as well as a way of structuring health care. It is also viewed as a core competence among health care professionals. PCC involves performing caring actions primarily focused on the person, in contrast to being solely task or disease oriented. The prerequisites for PCC are personal knowledge, experience and resources, as well as taking both physical and emotional needs into account. Shared decision making is described as a key aspect of PCC [1] and is important for ensuring that the care is experienced as person-centered [2,3]. Person-centeredness is usually defined as an ethical, humanistic and holistic perspective on care, characterized by respect for subjectivity, capability and personhood [3]. PCC is performed in the physical and psychosocial environment that constitutes the person-centered climate (PCCL). A PCCL is essential for the provision of PCC [4]. The physical environment, people’s actions and being in the environment as well as the organizational philosophy of care are integral features of the PCCL. Recognition of this fact enables caregivers to provide PCC [5]. Kristensson Uggla [6] describes a threefold disadvantage that patients experience when subjected to hospital care: (1) the institutional disadvantage of being at the bottom of the hierarchical ladder in the health care organization, (2) the existential disadvantage of having poor health or complicated illness and thus being vulnerable and (3) the cognitive disadvantage of lacking previous experience of the current illness or health situation. All three of the above are relevant in the context of advanced thoracic surgery. PCC is implemented to reduce patients’ disadvantage and involves respect for their personal needs, mutual agreements, patient participation and the perspective that the patient is more than her/his disease [7]. PCC, as a core competence among health care professionals, has been investigated previously and recently there has been emphasis on the PCCL [8,9,10]. The intention is to measure experiences among patients and their significant others.

### Framework

This study is based on the following framework proposed by McCormack et al. [11], suggesting a multi-professional approach to the development of PCC:

1. *Prerequisites:* The professionals must adopt characteristics such as professional conduct and interpersonal interaction skills. They must be engaged and aware of their own behavior.
2. *The care environment:* The context where the care takes place should involve a structure that supports shared power and decision making between the patient and the professionals. Different competencies are valued and there is an innovative atmosphere that encourages the testing of new strategies.
3. *Person-centered processes:* The caring actions stem from patients’ values and engagement. The professionals are close to the patients, not only satisfying basic needs, but working with shared decision making.
4. *Outcomes*: These include satisfaction with the care, participation, sense of well-being and the aim of creating a safe environment involving hospitality. In order to enable these outcomes, professional characteristics must be evident along with a context that supports person-centeredness.

**Figure 1:**
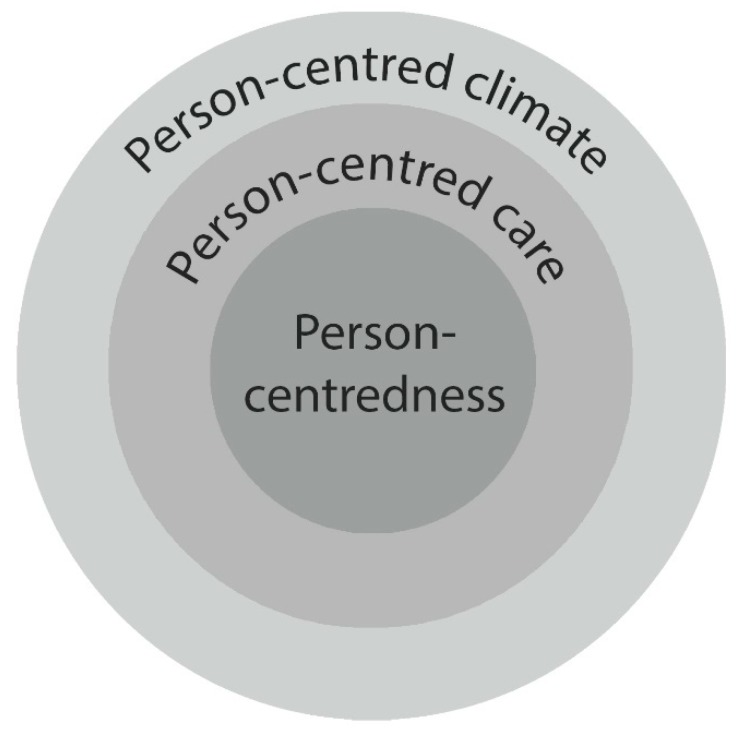
Interrelated layers of person-centeredness.

The PREM BTSQ-P was developed based on the understanding that person-centeredness consists of three different interrelated layers as illustrated in Figure 1 [12]. The core is the person-centeredness, i.e., being taken seriously, which is experienced by the patient when health care professionals provide person-centered interventions. The climate in which these interventions take place can be measured by the Person-Centered Climate Questionnaire (PCQ) [5]. This understanding was developed inductively by interviewing patients considered non-urgent by the Swedish Ambulance Service and therefore not conveyed to the Accident and Emergency Department (A&E). According to the patients, the essence of person-centeredness is being taken seriously [13]. An assumption is that the climate affects the possibility to provide PCC. The experience of what constitutes person-centeredness is purely subjective, but probably related to the climate in which the person-centered actions take place. However, this relationship requires elaboration.

As a patient-reported experience measure (PREM) in emergency and hospital care, being taken seriously is suggested to be a significant concept in the patient’s wish for PCC [10,13]. The meaning of taking patients seriously and the impact when this phenomenon is absent has previously been reported by Rantala, Ekwall and Forsberg [13]. Further research on the concept has resulted in a tentative PREM instrument, the Being Taken Seriously Questionnaire—Patient version (BTSQ-P) [10]. It was found that it is possible to define and measure being taken seriously by means of a one-factor solution covering eight items.

Highly specialized thoracic surgical care for patients suffering from life-threatening heart or lung conditions is characterized by its technically intense environment and rapid pace. High-tech caring environments involve a risk that the patient is not approached as a person due to the fact that the care is organized on the basis of signs, biomedical markers and medical diagnoses. In general, in-hospital caring environments today are highly technical and the advanced disease complexity in many patients together with the shortage of hospital beds means that high-tech care is expected to be delivered within a shorter period than was previously the case [14]. Patients in high-tech environments often feel strongly dependent on and subjected to the technical equipment. An unfamiliar and frightening environment can increase a patient’s experience of being objectified as opposed to being seen as a person [15]. In Swedish health care today, in thoracic surgical units, where it is challenging to create a PCCL and deliver PCC, no previous measurements have been performed.

Thus, the rationale behind this study is to further test whether the tentative BTSQ-P can serve as a solid PREM instrument in terms of validity and reliability in a high-tech hospital environment performing advanced thoracic surgery. At present, there is no other PREM instrument that measures person-centeredness in high-tech hospital environments. Therefore, the aim of this study was to describe the development and initial psychometric evaluation of a measure of person-centeredness by means of being taking seriously.

## 2. Materials and Methods

### 2.1. Setting

Patients undergoing advanced heart surgery, e.g., by-pass operations, valve replacement, aortic surgery and thoracic transplantation are admitted to the thoracic surgical ward (TSW). There are six single rooms and seven rooms with multiple beds where women and men are placed together. There is advanced technical surveillance with telemetry and other monitoring devices. The staff turnover was high during the data collection period (March–December 2018) when approximately 30 registered nurses and 30 assistant nurses worked in the TSW.

### 2.2. Participants and Procedures

In this cross-sectional survey, two questionnaires, the Being Taken Seriously Questionnaire—Patient version (BTSQ-P) and the Person-Centered Climate Questionnaire—Patient version (PCQ-P) were handed out during spring 2018 to patients undergoing heart surgery or thoracic transplantation at a Swedish University Hospital. The participants were included consecutively by an experienced thoracic nurse when in the TSW. Inclusion criteria were adult patients who had undergone heart surgery or thoracic transplantation, were mentally lucid, could understand Swedish and were admitted to the TSW for postoperative care. The sample size was based on existing recommendations for psychometrical studies. Thus, a sample-to-item ratio of 5:1 was in accordance with Suhr [16] and, to compensate for a potentially low response to the questionnaire, we also considered the recommendations by Barrett and Kline [17] regarding the item-to-sample ratio in psychometric studies. The latter approach allows for an item-to-sample ratio of 5:1 based on the reasoning that the item-to-sample ratio has no effect on factor stability [17]. Thus, 40 participants would have been sufficient to evaluate the psychometric properties of the eight-item BTSQ-P. However, we wanted to ensure excellent measurement properties and therefore oversampling was adopted. A total of 88 patients were invited to participate in the study and nine declined, mainly due to fatigue. One patient needed assistance when responding due to poor vision. The analyses were based on 79 thoracic patients, 55 men and 24 women with a mean age of 64 years (SD 12.8 years). The youngest patient was 33 years old and the oldest was 91 years old. The majority (*n* = 33) underwent Coronary Artery By-pass Grafting (CABG), followed by valve surgery (*n* = 17). There were three transplant recipients. The length of time as an in-patient was divided into three groups: <7 days (*n* = 20), >7 days (*n* = 35) and >three weeks (*n* = 24). The participants filled in the questionnaires before discharge and handed them back in a sealed envelope after giving their written informed consent.

### 2.3. The Instruments

#### 2.3.1. The Being Taken Seriously Questionnaire—Patient Version (BTSQ-P)

The development and testing steps are presented in Table 1. The lowest score was 6 and the maximum score was 48. When validated for the ambulance service, the factor loadings ranged from 0.836 to 0.962 and the internal consistency coefficient was 0.96 [10]. The instrument included the following eight items:

1. The staff listened to me,
2. I received help to understand what has happened,
3. I received help to understand what is about to happen,
4. My concerns have been taken seriously,
5. My symptoms have been taken seriously,
6. I have been taken seriously as a person,
7. The staff made me feel good in the present moment, and
8. The staff made me feel safe.

#### 2.3.2. The Person-Centered Climate Questionnaire—Patient Version (PCQ-P)

The Person-Centered Climate Questionnaire—Patient version (PCQ-P) was developed in order to ask patients about their preferences when being admitted to health care. It includes three subdimensions: climate of safety, climate of everydayness and climate of hospitality [5]. The response alternatives comprise a 6-point Likert scale ranging from 1 (No, I totally disagree) to 6 (Yes, I agree completely). *Climate of safety* concerns patients’ sense of safety in relation to the clarity, availability and competence of staff. The cleanliness of the physical environment is also important. *Climate of everydayness* pertains to whether the environment is more like a home than an institution. *Climate of hospitality* involves a feeling of confirmation, being welcomed and invited [5]. The PCQ score is shown in Table 2.

### 2.4. Data Analysis

The following analyses were performed stepwise using the IBM Statistical Package for the Social Sciences version 25 (SPSS^®^), Factor^®^ analysis software (Universitat Rovira i Virgili Tarragona, Tarragona, Spain) and R^®^ analysis software (Lucent Technologies, Murray Hill, NJ, USA):

1. A principal component analysis,
2. A parallel analysis based on minimum rank factor analysis [18],
3. An exploratory factor analysis,
4. Exploring relationships between the BTSQ-P and PCQ-P by means of Spearman’s correlation,
5. Standard multiple regression.

The BTSQ-P construct validity was investigated by means of corrected item–total correlations, i.e., the correlation between each item and the total score of the remaining items in the instrument. In order to eliminate weak and/or too narrow items, a lower cut-off limit of 0.3 and a higher cut-off limit of 0.7 were chosen. This agrees with Streiner et al. [19], who state that newly developed instruments should be checked for homogeneity and that a moderate correlation among items should be sought in relation to the total score of the instrument. The reliability of the BTSQ-P was assessed using ordinal alpha and Cronbach’s alpha, with preferred alpha values between 0.7 and 0.9. To evaluate whether the internal consistency could be improved by excluding items, the reliability analysis included the version of alpha if item is deleted. An alpha of 0.6 might be considered acceptable and therefore values between 0.6 and 0.7 were not automatically excluded without further consideration of the entire instrument’s alpha value [20,21].

### 2.5. Ethical Considerations

The ethical code of conduct was followed and conformed to the ethical guidelines adopted by the Swedish Research Council. Consent, confidentiality, utility and information were taken into account in line with the Declaration of Helsinki [22] and Swedish ethical protocols and legislation (SFS 2003:460). Each participant received written information about the study, in which it was stated that the voluntary return of the questionnaires, which contained no information that could identify the participants (i.e., not tracible), was considered consent to participate. The study was approved by the Swedish Ethical Review Board in Lund (Dnr 2018/383).

## 3. Results

### 3.1. Construct Validity and Reliability

The results from the principal component analysis (PCA) are presented in Table 3. The Kaiser–Meyer–Olkin test result was 0.797 and Bartlett’s test of sphericity was *p* ≤ 0.001. The one-factor solution accounted for 68% of the variance. We proceeded with an analysis of the multi-variate asymmetry skewness and kurtosis in accordance with Mardia [23]. The sample was significantly skewed (*p* ≤ 0.0001), with a skewness coefficient of 57.394 and kurtosis of 146.829. Thus, in order to enhance the precision, we performed an exploratory factor analysis (EFA) (Table 3) including polychoric correlation, resulting in a good Kaiser–Meyer–Olkin test result (0.834), Bartlett’s test of sphericity was *p* ≤ 0.00001 and interitem correlations ranged from 0.759 to 0.908, as shown in Table 3. The one-factor solution now accounted for 80.4% of the variance.

The internal consistency coefficient measured by Cronbach’s alpha was 0.87 and the ordinal alpha was 0.93.

### 3.2. Descriptive Statistics and Correlations

The median BTSQ-P was 46 (p25 = 41, p75 = 48) and the median PCQ-P sum score was 91.5 (p25 = 84, 25; p75 = 98) (min. 55, max. 102). The median for climate of safety was 58 (p25 = 53, 75; p75 = 60), where the lowest reported score for safety was 29. The median for climate of everydayness was 18 (p25 = 15, 25; p75 = 21) with 7 as the lowest reported score. In the dimension climate of hospitality, the median was 16 (p25 = 14; p75 = 18), with 8 as the lowest score. No gender differences were found in the two instruments and there were no differences between patients older or younger than 50 years. Finally, there were no differences related to the length of hospital stay.

A strong, significant correlation was identified between the BTSQ-P and climate of safety (rs = 0.633, *p* ≤ 0.001). There was a moderate, significant correlation between the BTSQ-P and climate of everydayness (rs = 0.549, *p* ≤ 0.001) as well as between the BTSQ-P and climate of hospitality (rs = 0.488, *p* ≤ 0.001).

### 3.3. Possible Explanatory Factors

The regression model (Table 4) explained 41.7% of the variance in the BTSQ-P (*p* < 0.0001) (R^2^ = 0.443 and adjusted R-Square = 0.417). The climate of Safety makes the strongest individual contribution ((*p* ≤ 0.001) to explaining the variance in the BTSQ-P (Beta.554) in contrast to climate of everydayness (Beta.242, *p* = 0.051) and climate of hospitality (Beta.048, *p* = 0.724). Preliminary analyses were conducted to ensure no violation of the assumptions of normality, linearity, multi-collinearity and homoscedasticity with tolerance > 0.10 VIF < 10 and Cook’s Distance < 1 (0.203).

## 4. Discussion

### 4.1. Discussions of the Findings

Patient-reported outcome measures (PROMs) and PREMS are necessary to determine whether health services also lead to better health from patients’ perspectives. The patients in this study experienced the care related to cardiac surgery as person-centered due to being taken seriously and perceiving a climate of safety. The strong relationship between the BTSQ-P and climate of safety was expected because it is often argued that safe care depends on person-centeredness. Overall, the relationship between the BTSQ-P and the PCQ-P support the hypothesis that person-centeredness consists of three different interrelated layers as illustrated in Figure 1. The construct validity improved when performing the factor analysis and the variance increased to over 80%. Thus, the BTSQ-P seems to be a valid, reliable and easy to use PREM, covering most of the variance of experiences within highly specialized in-hospital care. Combining the BTSQ-P with existing PROMs might be a useful approach in the future to evaluate the impact of thoracic surgical care.

The available PROMs are mainly generic and disease specific, with demonstrated validity and reliability for ascertaining patient’s self-rated health and health-related quality of life in terms of physical, social and mental well-being as well as functional ability [24]. There is a demand for increased use of such instruments in clinical routines as well as in national quality registers, and thus we argue that, based on the finding in this study, the BTSQ-P is a valuable contribution to in-hospital care, despite the fact that it needs further testing in different in-hospital settings. However, more effective ways of collecting data than paper-based questionnaires must be developed. We suggest that the eight items can easily be transferred to a digital or mobile technical solution that enables continuous measurements over time to ensure stability in the provision of person-centered care. This also enables different language versions, where the next step will be to translate BTSQ-P into other languages.

There are inherent challenges when using PREMs as the satisfaction with care might be influenced by expectations, personality traits and previous care experiences. One way to minimize the impact of these factors is to ask about specific experiences, e.g., the perioperative care in relation to heart surgery. The feedback on the measurement can be more easily adjusted when using an electronic health solution than traditional methods and the findings can be used directly in the encounter with the patient to facilitate mutual understanding and partnership. It has been shown that systematic use of patient-reported measures improves and facilitates the communication and relationship between patients and health care professionals. Health problems are identified and dealt with in a more proactive way [25]. From a patient perspective, this is highly important [26,27,28], but it requires a will and readiness to collect and analyze the measurements in order to enable quality improvements.

### 4.2. Methodological Considerations

According to Aaronson et al. [29], an instrument that is suitable for evaluating a target population should fulfil the following eight requirements: provide a conceptual and measurement model, demonstrate reliability, validity, responsiveness and interpretability, involve reasonable respondent and administrative burden, as well as facilitate alternative forms, and cultural and language adaptations [29]. Other researchers have defined similar, but less comprehensive, quality criteria [30,31]. When developing a new questionnaire, not only should the evaluation of validity and reliability be provided, but also a clear description of the measurement aims, target population, concept of interest (theoretical framework), item selection, item reduction and the work required from respondents [32]. We believe that the BTSQ-P fulfils the above-mentioned quality criteria except for reliability, which is not yet fully tested.

## 5. Conclusions

Being taken seriously might be a generic key factor leading to a sense of being empowered and restored as a person when in high-tech in-patient settings. The newly developed instrument called the Being Taken Seriously Questionnaire—Patient version offers a possibility to include person-centeredness as a PREM measure, which is in line with current demands to focus on the patient perspective of care both in Swedish health care and all over Europe.

## Figures and Tables

**Table 1:** The development and testing steps of the Being Taken Seriously Questionnaire—Patient version (BTSQ-P).

Step	Task *	Performance
1	Specificying measurment goals	The BTSQ-P was developed for adult persons undergoing health care. They should be able to read and write Swedish or any other language into which the instrument is translated. The primary purpose of the instrument is to be discriminative and evaluative, serving as a generic patient-reported experience measures (PREMs). The instrument is supposed to be self-administered, but is also suitable for telephone interviews.
2	Item generation	The item pool was chosen from open-ended, in-depth interviews (as described by Rantala, Ekwall and Forsberg [13]), review of the specific literature on person-centered care and discussions with health care professionals.
3	Item reduction	No item reduction was performed. The original eight items were retained.
4	Questionnaire formatting	Words were used that apply to the widest range of cultures and geographic areas in order to facilitate translation and widespread use. Each response relates to the perceived importance of the BTSQ-P construct on a 6-point Likert-type scale ranging from complete disagreement (1) to complete agreement (6).
5	Pretesting	A small pretest (as part of the face validity assessment) was performed involving five thoracic patients.
6	Reliability	Scale reliability was estimated using the ordinal alpha as well as Cronbach’s alpha. Reliability will be further tested in future research.
7	Validity	The content validity was assessed in two ways: comparison with the literature on person-centeredness and consultation with an expert group comprising two senior nurse researchers (with experience of scale development) and two clinical specialist nurses with specific interest in and knowledge of PCC. They were asked to assess the relevance, clarity and readability of the items. The experts considered the content validity good and that the items reflect what the literature describes as central aspects of person-centeredness. In addition, a content validity index was evaluated by eight patients and found satisfactory, in terms of the relevance, clarity and readability of the items. Construct validity was determined by the confirmatory multi-trait analysis program and by explorative principal component analysis (with oblique, varimax rotation) as well as by examining the relation between the BTSQ-P and the generic Person-Centered Climate Questionnaire (PCQ-P) instrument.
8	Interpretability	We use the so-called anchor-based approach, where the changes in BTSQ-P measures are compared or anchored to other clinically meaningful outcomes. This will be tested further.

* Tasks 1–4 relate to development and tasks 5–8 to the test phase.

**Table 2:** The minimum and maximum score in the Person-Centered Climate Questionnaire—Patient version (PCQ-P) and subdimensions.

	Min. Score	Max. Score
PCQ-P, the whole instrument	17	102
A climate of safety (items 1–10)	10	60
A climate of everydayness (items 11–14)	4	24
A climate of hospitality (items 15–17)	3	18

**Table 3:** Construct validity based on both a principal component analysis (PCA) and an exploratory factor analysis (EFA) of the eight items in the Being Taken Seriously Questionnaire—Patient version (BTSQ-P).

Items	Factor Loadings PCA	Factor Loadings EFA
The staff listened to me	0.697	0.802
I received help to understand what has happened	0.684	0.806
I received help to understand what is about to happen	0.718	0.821
My concerns have been taken seriously	0.731	0.863
My symptoms have been taken seriously	0.801	0.908
I have been taken seriously as a person	0.806	0.875
The staff made me feel good in the present moment	0.759	0.870
The staff made me feel safe	0.636	0.759

**Table 4:** Linear regression model with the PCQ-P subscales, climate of safety, climate of everydayness and climate of hospitality, as independent variables and the BTSQ-P as a dependent variable. The person-centered climate accounts for 41.7% (adjusted R^2^) of the BTSQ-P.

Independent Variable	B	Beta	t	*p*
Climate of safety	0.383	0.554	4.793	≤0.001
Climate of everydayness	0.252	0.242	1.986	0.051
Climate of hospitality	−0.085	0.048	−0.354	0.724

R^2^ = 0.443; adjusted R^2^ = 0.417; B = unstandardized regression coefficient; Beta = standardized regression coefficient. PCQ-P = Person-Centered Climate Questionnaire—Patient version; BTSQ-P = Being Taken Seriously Questionnaire—Patient version.
